# Post synthetic exchange enables orthogonal click chemistry in a metal organic framework†
†Electronic supplementary information (ESI) available: Experimental details, linker syntheses, ^1^H and ^19^F NMR spectra, PXRD patterns, IR spectra, and SEM images. See DOI: 10.1039/c8dt04563a


**DOI:** 10.1039/c8dt04563a

**Published:** 2018-11-28

**Authors:** Ulrike Fluch, Brian D. McCarthy, Sascha Ott

**Affiliations:** a Department of Chemistry , Ångström Laboratory , Uppsala University , Box 523 , 75120 Uppsala , Sweden . Email: sascha.ott@kemi.uu.se

## Abstract

Biphenyl-4,4′-dicarboxylic acid derivatives containing either azide or acetylene functional groups were inserted into UiO-67 metal organic frameworks (MOFs) *via* post synthetic linker exchange. Sequential and orthogonal click reactions could be performed on these modified MOFs by incubating the crystals with small molecule substrates bearing azide or acetylene groups in the presence of a copper catalyst. ^1^H NMR of digested MOF samples showed that up to 50% of the incorporated linkers could be converted to their “clicked” triazole products. Powder X-ray diffraction confirmed that the UiO-67 structure was maintained throughout all transformations. The click reaction efficiency is discussed in context of MOF crystallite size and pore size. As the incorporation of clicked linkers could be controlled by post synthetic exchange, this work introduces a powerful method of quickly introducing orthogonal modifications into known MOF architectures.

## Abstract 2

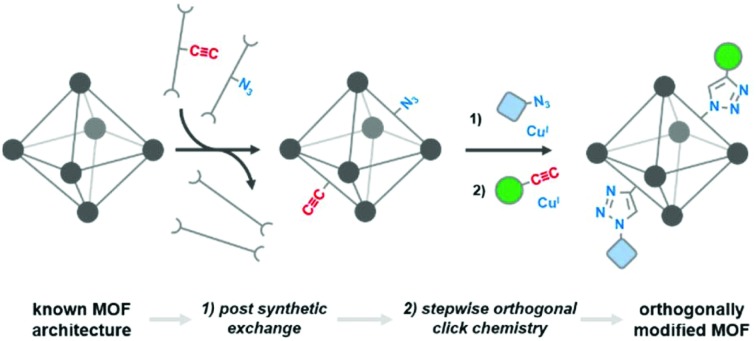
Post synthetic linker exchange can be combined with Cu-catalyzed alkyne/azide click chemistry to enable orthogonal modification of known metal organic frameworks.

## Introduction

Metal organic frameworks (MOFs) have moved beyond ‘simple’ homogeneous materials into complex multiple-domain structures.^1^–^6^ Given the enormous number of applications proposed for MOFs,^7^–^11^ there is a clear need for simple and robust methods to quickly add functionality to MOFs.

One desirable route for rapidly adding functionality is by coupling known MOF syntheses with unrelated yet well-established synthetic paradigms. We envisioned that post synthetic exchange^12^ of linkers bearing extra reactive groups could be combined with MOF click chemistry^13^–^21^ to allow *orthogonal* MOF modification. Orthogonal linker modification *via* a selective and stepwise tuning of specific subsets of linkers within a MOF would rapidly permit the construction of multi-functional MOFs. The vast majority of post synthetic MOF modifications methods do not discriminate within the target. For example, post synthetic metal ion exchange^22^,^23^ and post synthetic linker exchange usually affect all accessible exchange sites. One ‘exception’ to this rule is core–shell post synthetic modification arising from slow diffusion or steric hindrance.^24^ Given the relative lack of methods for through-MOF orthogonal modification, a clear need exists for new simple robust methods.

Orthogonal click chemistry is generally not possible through routine solution chemistry. If a mixture of substrates containing either acetylene or azide groups is exposed to catalytic conditions and a click reaction partner, the expected result is a statistical mixture of product and substrate cross-coupling. We expect that if two different click substrates are immobilized within the same MOF framework this unproductive cross-reaction can be avoided and so allow selective stepwise click reactions on the same material.

Very recently orthogonal copper(i)-catalyzed azide–alkyne cycloaddition (CuAAC) click chemistry within a MOF has been shown using a MOF built entirely of linkers bearing azide and acetylene functional groups. After synthesis, this UiO-68 (UiO = University of Oslo) type MOF could undergo sequential click reactions within the solid crystals in quantitative yield.^25^ This method is clearly useful, though limited to click reaction partners capable of surviving solvothermal synthesis.

**Fig. 1:**
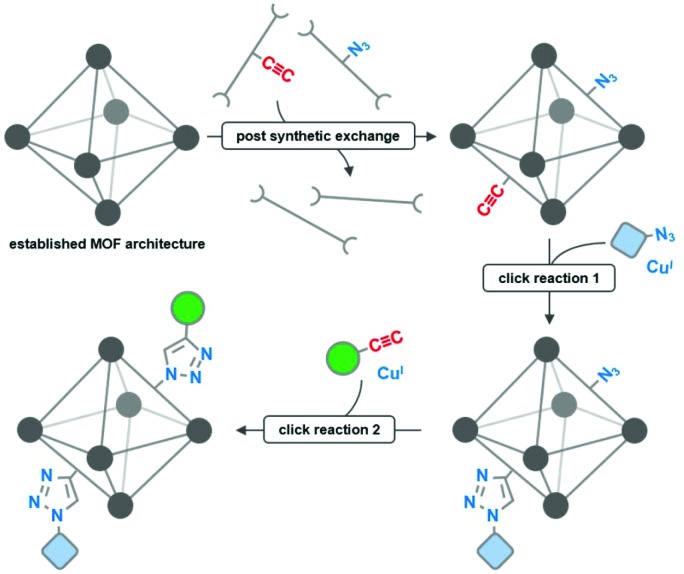
Targeted route for achieving orthogonal and sequential click reactions in a MOF *via* post synthetic exchange of a fraction of the original linkers for linkers bearing azide and acetylene groups, followed by two click reactions.

Consequently, we developed a new orthogonal modification scheme by synthesizing a known MOF followed by post synthetic exchange of a *fraction* of the linkers with linkers containing azide or acetylene groups. Following post synthetic exchange, CuAAC click reactions could be performed sequentially and orthogonally by introducing one click partner at a time (Fig. 1).

Using post synthetic exchange provides three unique advantages: (1) control over the final fraction of modified – and so clicked – linkers, (2) permitting the use of known MOFs, and (3) providing a way to introduce reactive sites that would not survive the initial MOF solvothermal synthesis. The last point is especially important, as it has been shown that the azide-functionalized linker we used here does not survive typical UiO-67 solvothermal conditions.^26^

## Results and discussion

UiO-67 (constructed from [1,1′-biphenyl]-4,4′-dicarboxylic acid) was chosen for this study due to its good bulk structural stability, relatively large pore size, and proven ability to engage in post synthetic linker exchange reactions.^27^,^28^

**Fig. 2:**
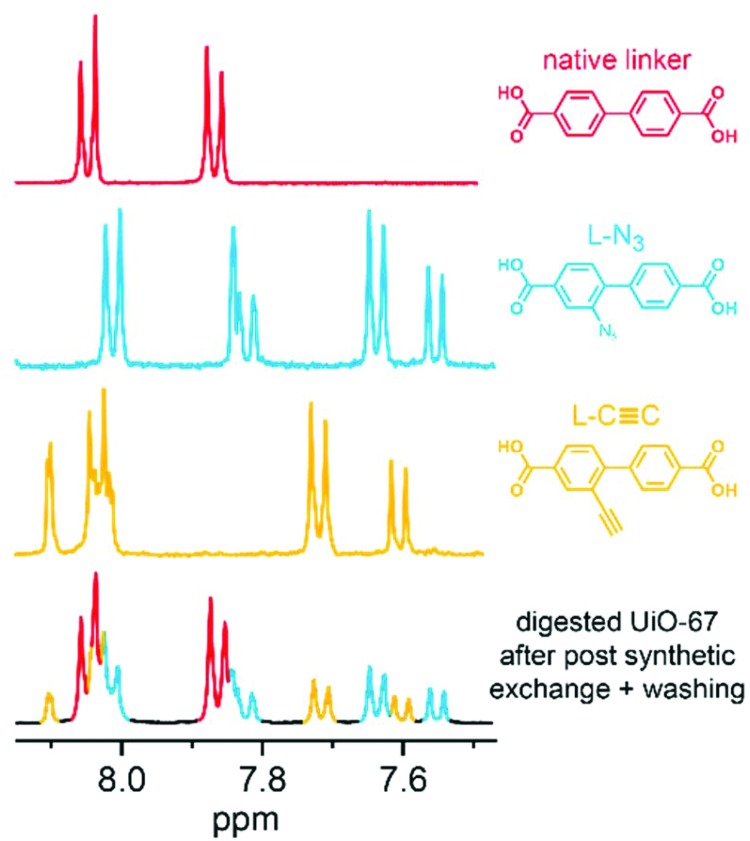
^1^H NMR spectra in d_6_-DMSO of native UiO-67 linkers, azide and acetylene modified ligands L-N_3_ and L-C≡C, and UiO-67 MOF after post synthetic exchange, washing, and digestion.

Conditions were first explored for post synthetic exchange of the native linkers with azide and acetylene functionalized linkers. Suspensions of 10 mg UiO-67 in 2 mL of 3 : 2 : 1 v/v solutions of THF : MeOH : H_2_O were prepared. To these were added [1,1′-biphenyl]-4,4′-dicarboxylic acid functionalized with either an azide or acetylene group (L-N_3_ and L-C≡C respectively, as shown in Fig. 2) and the suspension stirred for 24 hours. After exchange, the solids were collected by centrifugation, washed at least ten times with clean solvent, and then digested in d_6_-DMSO with aqueous HF for ^1^H NMR quantification (see ESI† section “Post synthetic linker exchange”). Powder X-ray diffraction (PXRD) confirmed that the UiO-67 structure was maintained after post synthetic exchange (Fig. S11†) while IR spectroscopy confirmed the presence of azide and acetylene functional groups (Fig. S12†).

Table 1 summarizes the linker exchange efficiencies under different exchange conditions. Greater than 40% of the native linkers could be exchanged with the azide-functionalized linker L-N_3_ at room temperature, while exchange was lower for the acetylene linker L-C≡C. Increasing the temperature to 40 °C did not appreciably change the incorporation yield for the azide linker. No thermocyclization of the azide linker, as seen for solvothermal synthesis of UiO-67 containing L-N_3_,^26^ was observed.

**Table 1:** Post synthetic exchange incorporation percentages of [1,1′-biphenyl]-4,4′-dicarboxylic acid functionalized with azide (L-N_3_) or acetylene (L-C≡C) into 10 mg of UiO-67 suspended in 2 mL of 3 : 2 : 1 v/v solutions of THF : MeOH : H_2_O after 24 hours of stirring

*T*	Linker concentration^*a*^		Incorporation^*b*^	
	L-N_3_	L-C≡C	L-N_3_	L-C≡C
40 °C	20 mM	—	41%	—
RT	20 mM	—	43%	—
RT	—	20 mM	—	36%
RT	13 mM	13 mM	33%	26%

^*a*^Relative to the calculated total number of native linkers in 10 mg of dried UiO-67.

^*b*^The percentage of total linkers which were exchanged as determined by ^1^H NMR of digested MOFs after post synthetic exchange, washing, and drying under vacuum.

Crucially, both linkers could be introduced into the same UiO-67 material through this post synthetic exchange strategy (Table 1). With 13 mM of both linkers in solution, the incorporation of L-N_3_ and L-C≡C were 33% and 26%, respectively. These exchange yields track with the lower incorporation of L-C≡C observed in the single substitution experiment. Fig. 2 shows a typical ^1^H NMR of UiO-67 digested after simultaneous post synthetic exchange with both L-N_3_ and L-C≡C.

As a MOF containing large numbers of modified linkers may result in excessive steric crowding during subsequent click reactions, we sought to generate UiO-67 with fewer substituted linkers. Indeed, a straightforward decrease in the net equivalents of both linkers present during exchange (1.1 equivalents of each linker, 10 mM concentration) yielded UiO-67 with *ca.* 10% modified linkers with either L-N_3_ or L-C≡C. UiO-67 containing *ca.* 10% of modified linkers were used in subsequent click reaction experiments.

Test CuAAC click reactions on these MOF materials were performed with the copper click catalyst [Cu(CH_3_CN)_4_]PF_6_ and the fluorine-labeled substrates 1-ethynyl-4-(trifluoromethyl)benzene and 4-azido-1,1,1-trifluorobutane. ^19^F NMR was used to confirm that the MOF material after the click reactions contained the corresponding triazole products (ESI Fig. S7–S10†). ^1^H NMR was also used to assess if the expected click products were present and to quantify the click reaction yields by comparison with the unreacted linkers (see ESI section “Click reactions and NMR spectra” and Fig. S1–S6†).

Click reactions were performed under inert atmosphere at 50 °C in freshly distilled THF for 24 hours. In general, 20 mg of functionalized UiO-67 was mixed with 0.5 molar equivalent of [Cu(CH_3_CN)_4_]PF_6_ (calculated on the number of clickable functionalities in the MOF) in 1.5 ml THF and degassed for 5 minutes prior to the addition of the fluorinated click partners (2 eq. based on the number of reactive functional groups).

Control reactions (Table 2A) established that while no click chemistry occurred without both substrates and copper catalyst, single click reactions could be successfully performed. The coupling of the azide-containing UiO-67 with an acetylene click partner was found to proceed to 46% completion. Conversely, coupling of the acetylene-containing UiO-67 with an azide click partner reached 59% conversion in the same time.

**Table 2:** Click reactions of UiO-67 and UiO-67 modified by post synthetic exchange to include L-N_3_ and L-C≡C appended linkers. Clicked products detected by ^1^H NMR of post-reaction digested MOF

(A) Single click reactions				
Linkers	Cu^I^?	Reactants	Observed clicked products	Yield
Native only	Y	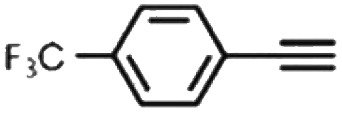	None	—
L-N_3_	Y	—	None	—
L-N_3_	N	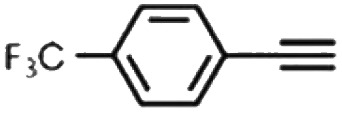	None	—
L-N_3_	Y	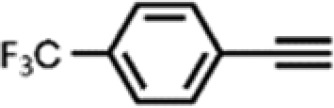	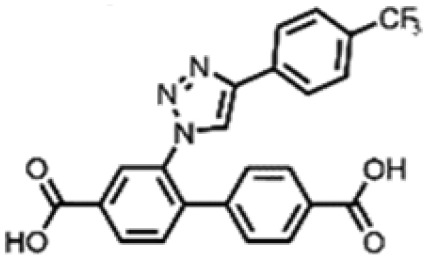	46%
L-C <svg xmlns="http://www.w3.org/2000/svg" version="1.0" width="16.000000pt" height="16.000000pt" viewBox="0 0 16.000000 16.000000" preserveAspectRatio="xMidYMid meet"><metadata> Created by potrace 1.16, written by Peter Selinger 2001-2019 </metadata><g transform="translate(1.000000,15.000000) scale(0.005147,-0.005147)" fill="currentColor" stroke="none"><path d="M0 1760 l0 -80 1360 0 1360 0 0 80 0 80 -1360 0 -1360 0 0 -80z M0 1280 l0 -80 1360 0 1360 0 0 80 0 80 -1360 0 -1360 0 0 -80z M0 800 l0 -80 1360 0 1360 0 0 80 0 80 -1360 0 -1360 0 0 -80z"/></g></svg> C	Y	—	None	—
L-C <svg xmlns="http://www.w3.org/2000/svg" version="1.0" width="16.000000pt" height="16.000000pt" viewBox="0 0 16.000000 16.000000" preserveAspectRatio="xMidYMid meet"><metadata> Created by potrace 1.16, written by Peter Selinger 2001-2019 </metadata><g transform="translate(1.000000,15.000000) scale(0.005147,-0.005147)" fill="currentColor" stroke="none"><path d="M0 1760 l0 -80 1360 0 1360 0 0 80 0 80 -1360 0 -1360 0 0 -80z M0 1280 l0 -80 1360 0 1360 0 0 80 0 80 -1360 0 -1360 0 0 -80z M0 800 l0 -80 1360 0 1360 0 0 80 0 80 -1360 0 -1360 0 0 -80z"/></g></svg> C	N	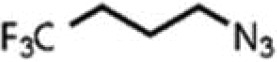	None	—
L-C <svg xmlns="http://www.w3.org/2000/svg" version="1.0" width="16.000000pt" height="16.000000pt" viewBox="0 0 16.000000 16.000000" preserveAspectRatio="xMidYMid meet"><metadata> Created by potrace 1.16, written by Peter Selinger 2001-2019 </metadata><g transform="translate(1.000000,15.000000) scale(0.005147,-0.005147)" fill="currentColor" stroke="none"><path d="M0 1760 l0 -80 1360 0 1360 0 0 80 0 80 -1360 0 -1360 0 0 -80z M0 1280 l0 -80 1360 0 1360 0 0 80 0 80 -1360 0 -1360 0 0 -80z M0 800 l0 -80 1360 0 1360 0 0 80 0 80 -1360 0 -1360 0 0 -80z"/></g></svg> C	Y	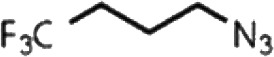	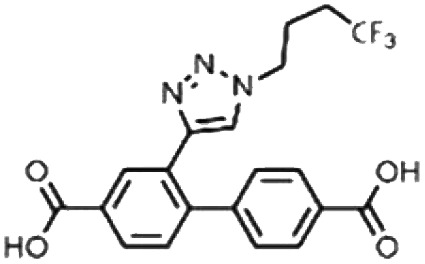	59%
L-N_3_ & L-C≡C	Y	—	None	—

(B) Double click reaction				
Linkers	Step	Reactants	Observed clicked products	Yield
L-N_3_ & L-C <svg xmlns="http://www.w3.org/2000/svg" version="1.0" width="16.000000pt" height="16.000000pt" viewBox="0 0 16.000000 16.000000" preserveAspectRatio="xMidYMid meet"><metadata> Created by potrace 1.16, written by Peter Selinger 2001-2019 </metadata><g transform="translate(1.000000,15.000000) scale(0.005147,-0.005147)" fill="currentColor" stroke="none"><path d="M0 1760 l0 -80 1360 0 1360 0 0 80 0 80 -1360 0 -1360 0 0 -80z M0 1280 l0 -80 1360 0 1360 0 0 80 0 80 -1360 0 -1360 0 0 -80z M0 800 l0 -80 1360 0 1360 0 0 80 0 80 -1360 0 -1360 0 0 -80z"/></g></svg> C	(1)	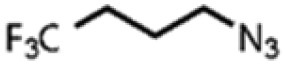	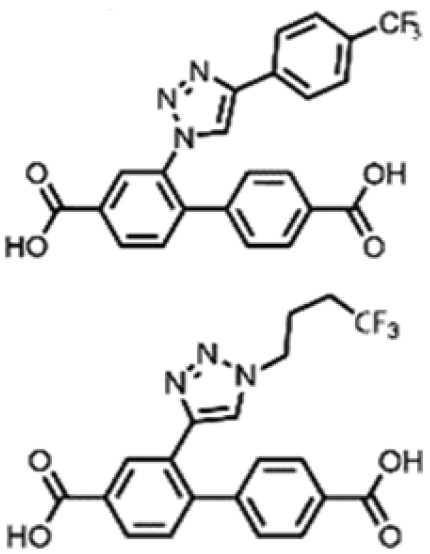	50%
	(2)	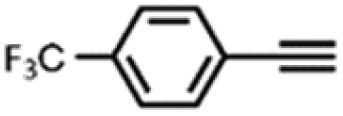		50%

Next, control reactions (Table S1†) established that orthogonal click reactions could be performed on UiO-67 containing both azide and acetylene modified linkers. The order in which the click partners were introduced was not observed to change the outcome. Again, no click chemistry occurred if either substrate or copper catalyst was absent, and no thermocyclization of L-N_3_ was seen.

In each case when copper catalyst was present, the final MOF material remained slightly blue even after extensive washing with neat solvent and solvent containing EDTA, indicating that some copper remains trapped inside the MOF pores. Quantification by ICP of the copper impurity after a double click reaction performed on UiO-67 containing *ca.* 10% of both modified linkers found that the ratio of zirconium to copper was *ca.* 27 : 1, a relatively minor amount. Finally, ^1^H NMR found that the unreacted linkers did not lose their azide and acetylene functional groups.

Given the difficulty in quantifying click yields on small batches of UiO-67, click yield efficiency was rigorously quantified by ^1^H NMR using a larger batch of UiO-67 containing *ca.* 80% native ligand and *ca.* 10% of each of the azide and acetylene ligands. Sequential click reactions using 10 eq. of each fluorinated substrate resulted in 50% click conversion of each modified linker (Table 2B). The remaining unreacted linkers maintained their azide and acetylene functional groups as found by ^1^H NMR. PXRD confirmed that the MOF material after the click reactions was still the UiO-67 structure (Fig. S11†). Scanning electron microscopy indicated that some superficial crystal damage occurs (Fig. S13–S20†), likely the result of mechanical damage from stirring. Shaking the samples instead of stirring during the click reactions appeared to decrease crystal damage (*e.g.*, comparison of Fig. S19 and S20†).

Surface area analysis by N_2_ sorption at 77 K was performed after sequential click reactions on UiO-67 containing both modified linkers. The BET surface area and the total pore volume of the MOF after click chemistry was found to be 1113 m^2^ g^–1^ and 0.50 cm^3^ g^–1^. As expected, this is lower than native UiO-67 MOF (2400 m^2^ g^–1^ for BET surface area and 0.91 cm^3^ g^–1^ for total pore volume).^27^,^29^,^30^ This significantly lower surface area and pore volume supports the hypothesis that the click reaction occurred not only at the MOF exterior surface – as seen in the surface-selective click chemistry found using UiO-66 bearing smaller pore windows^31^,^32^ – but also within the MOF interior The copper remaining inside the MOF after the double click reaction (see above) was in a relatively minor amount and is not believed to be a primary contributor to the loss of surface area.

Taken together, these results demonstrate that post synthetic exchange can be used to introduce linkers bearing either azide or acetylene functional groups into known MOFs. Stepwise and orthogonal click chemistry can then be performed in decent yields while maintaining the original bulk crystallinity.

To our knowledge this is the second only example of orthogonal click chemistry within a MOF,^25^ and the first to combine this strategy with post synthetic linker exchange. As the orthogonal click strategy used by Zhang *et al.*^25^ differed from that used herein, it is useful to compare the two methods.

The UiO-68 type MOF used by Zhang *et al.* was built entirely from linkers bearing azide or acetylene groups, while linkers with these reactive groups were introduced herein *via* post synthetic exchange into UiO-67. The PSE strategy on UiO-67 allows the preparation of MOFs with substantially fewer click reactive sites that could be useful in applications requiring fractional modification. However, the approximately 50% click conversion yields in this work is sharply contrasted by the 100% click conversion observed in Zhang *et al.*'s UiO-68 type framework.

The difference in click conversion yields between this work and that of Zhang *et al.* can be assessed based on relative pore sizes, click reaction partner sizes, reaction conditions, and macroscopic crystallite size. The triangular pore openings of UiO-67 (this study) and UiO-68 are *ca.* 8 and 10 Å, respectively,^27^ whereas the ethynylbenzene/(azidomethyl)benzene click partners used in the UiO-68 experiments are somewhat smaller than the click partners used here. Click reactions here were performed at 50 °C in THF for 24 hours, with 0.5 eq. of [Cu(CH_3_CN)_4_]PF_6_ catalyst, and 2 equivalents of click partners; in Zhang's work the reaction was carried out at 60 °C in DMF with CuI catalyst for 24 hours and unknown click partner stoichiometry.

Individual crystallite size also matters: smaller crystals should facilitate access to interior click reaction sites. To our knowledge, there is no rigorous study available comparing post synthetic exchange into different sized crystals. However, one comparison can be made between two papers that both used UiO-66. In one study, the incorporation of benzene-2,3,5,6-*d*_4_-1,4-dicarboxylic acid (H_2_BDC-*d*_4_) into *ca.* 100 μm crystals of UiO-66 was examined and a pronounced core–shell structure was found, with exchange only occurring in the outer layers.^24^ Conversely, for exchange of the bulkier 2-iodobenzene-1,4-dicarboxylate into <1 μm sized UiO-66 crystals uniform exchange is seen.^33^

The difference in crystallite size for this study and Zhang's UiO-68 work is not so pronounced. Here, crystals were *ca.* 1–3 μm across (*via* SEM, see Fig. S13–S20†), whereas the UiO-68 study appears to have consisted of larger crystals tens of micrometers wide.

Given these differences, a quantitative comparison is not straightforward; however, we suspect that the larger pore sizes of UiO-68 were the primary factor contributing to the reported quantitative click yields. Future studies comparing identical click reaction conditions using UiO-67 and UiO-68 crystals of similar size could address this question.

## Conclusions

MOFs have the potential to revolutionize industrial material science; as such, interest in new methods of functionalizing MOFs remains high. As there are thousands of MOF architectures to choose from, methods to easily modify *known* MOF materials are of special interest.

Post synthetic exchange is firmly established as a reliable method of tuning known MOFs,^23^,^34^ whereas click chemistry has shown its power in the world of molecular synthetic chemistry^35^,^36^ and within MOFs.^13^–^20^ *Via* the combination of these two methods, this work demonstrates a new approach to *orthogonally* modify MOFs. First, post synthetic exchange reactions showed that the native linkers in UiO-67 could be replaced with linkers bearing azide or acetylene groups. Stepwise incubation with a copper catalyst and small molecule click partners allowed the formation of two different triazole click products in a selective and orthogonal manner.

Control reactions established unambiguously that no click reactions occur if either the click partner or Cu catalyst is omitted. Quantification of click reaction yields *via*^1^H NMR revealed that up to 50% of the modified linkers could be converted to their clicked partners. PXRD analysis confirmed that the parent UiO-67 structure was maintained throughout all transformations.

Given the versatility of post synthetic exchange and power of click chemistry, we expect that this method of modifying MOFs in a stepwise and orthogonal fashion will be broadly useful. The ability to selectively change two different subsets of linkers within a MOF opens new avenues for rapid construction of complex functionality. From a fundamental perspective, the ability to selectively perform click chemistry on only one substrate while both azide and acetylene substrates are present is not normally possible in solution chemistry – it is through immobilization within a MOF framework that selective and non-statistical products can be obtained. Interesting questions remain regarding optimization of the click reaction efficiency, especially for MOFs with small pore diameters.

## Author contributions

UF performed research; UF and SO designed research and analysed data; BDM, UF, and SO wrote the paper.

## Conflicts of interest

There are no conflicts to declare.

## Supplementary Material

Supplementary information
