# Supplementary material for: Post synthetic exchange enables orthogonal click chemistry in a metal organic framework
Source: Dalton Trans. 2018 Nov 28;48(1):45–9. doi: 10.1039/c8dt04563a (PMC6336147; doi:10.1039/c8dt04563a)
Supplement: Supplementary file 1 [file DT-048-C8DT04563A-s001.pdf]

Electronic Supplementary Material (ESI) for Dalton Transactions.
This journal is © The Royal Society of Chemistry 2018

Supplementary Information for

Post synthetic exchange enables orthogonal click chemistry in a metal organic framework

Ulrike Fluch, Brian D. McCarthy, and Sascha Ott*

Department of Chemistry

Ångström Laboratory, Uppsala University

Box 523, 75120 Uppsala, Sweden

*Email: sascha.ott@kemi.uu.se

Table of Contents

General details	2
Synthetic details	3-9
Post synthetic linker exchange	10
Click reactions and NMR spectra	11-20
Powder X-ray diffraction patterns	21
Infrared spectroscopy	22
Scanning electron microscopy	23-25
References	26

General details

All purchased chemicals were used without further purification except where otherwise stated. Dimethylformamide used for synthesizing UiO-67 was pre-dried over 4 Å molecular sieves. X-ray powder diffraction patterns were collected using a Siemens D5000 utilizing a monochromatic Cu K_α radiation source at 40 kV, 40 mA for Cu Kα, (λ = 1.5406 Å) with a scan speed of 0.10 sec/step from 5 to 40º at a step size of 0.02º. NMR spectra were obtained using a JEOL Eclipse+ 400 MHz spectrometer and chemical shifts reported vs. the residual NMR solvent peak.[1] Infrared spectra (IR) were collected on a PerkinElmer ATR-FT-IR spectrometer. Scanning electron microscopy (SEM) was performed on a Zeiss 1550 with AZtec EDS equipped with InLens, SEII, and BSD detectors. The measurements were performed at 3 kV at working distances of 3.2 to 4.0 mm. The samples were sputtered prior to the measurements with a Polaron Sputter Coater (Au/Pd) for 40 seconds.

UiO–67 was synthesized according to known procedures.[2] In brief, 1 eq of [1,1'-biphenyl]-4,4'-dicarboxylic acid, 1 eq of $ZrCl_4$ and 33 eq of acetic acid were suspended in dry DMF (4 mL for 0.11 mmol ligand), placed in screw cap vials and heated at 120 °C for 24 hours. After cooling to room temperature, the clear supernatant was removed and the MOF crystals resuspended in fresh MeOH. The solvent was renewed after 24 hours with fresh solvent and this washing repeated three times, with the final MOF product stored in fresh MeOH.

Synthetic details

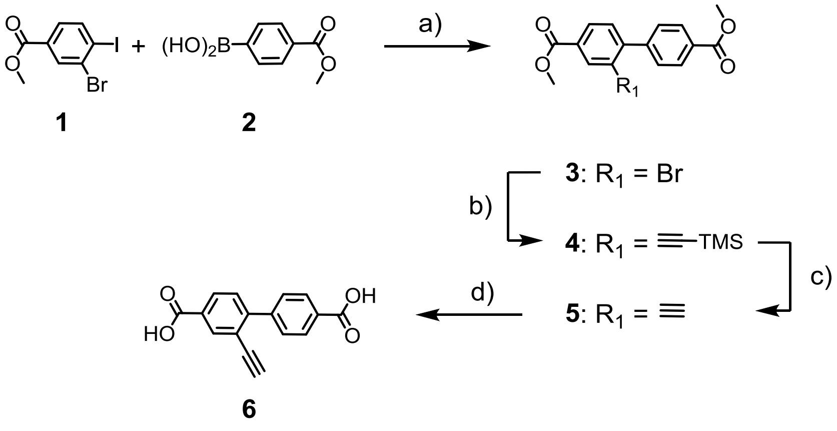

Scheme S1. Synthetic route to L-C≡C (compound **6**).

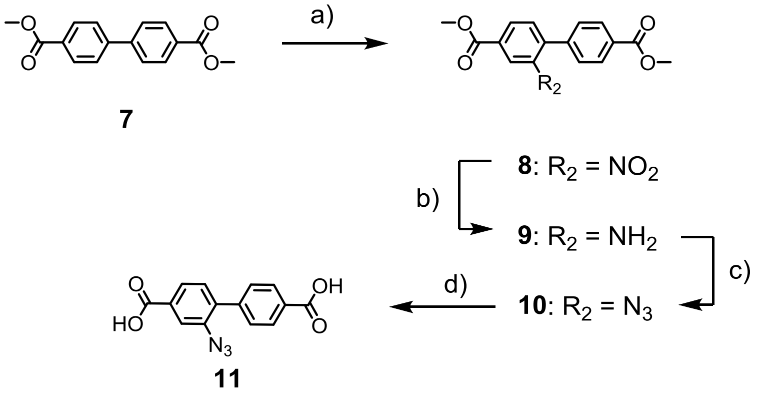

Scheme S2. Synthetic route to L-N_3 (compound **11**).

Synthesis of methyl 3-bromo-4-iodobenzoate (1):

1 g 3-bromo-4-iodobenzoic acid (1 eq) was suspended in 30 mL of dichloromethane. 889 µl (4 eq) of $SOCl_2$ was added dropwise and the reaction mixture stirred for 1 hour at room temperature. The mixture was cooled to 0 °C before 10 ml methanol was slowly added. After the addition was finished the reaction was warmed to room temperature and stirred overnight. The volatile components were removed and the residue redissolved in dichloromethane and water, extracted with dichloromethane, dried over $MgSO_4$ and the solvent removed under reduced pressure. 1.2 g of the product was isolated as brown solid (97% yield).

^{1}H NMR (400 MHz, CHLOROFORM-D) δ 8.23 (s, 1H), 7.94 (d, *J* = 8.1 Hz, 1H), 7.60 (d, *J* = 7.9 Hz, 1H), 3.91 (s, 3H).

^{13}C NMR (101 MHz, CHLOROFORM-D) δ 165.51, 140.55, 133.47, 131.64, 130.18, 129.04, 107.78, 100.02, 52.68.

Synthesis of dimethyl 2-bromo-[1,1'-biphenyl]-4,4'-dicarboxylate (3):

(4-(methoxycarbonyl)phenyl)boronic acid (400 mg, 1 eq) and **1** (211 mg, 1 eq) were dissolved in 10 ml DMF. Na_2CO_3 (248 mg, 2 eq) and 2 ml of water were added and the resulting mixture was degassed by bubbling nitrogen through the solution for 10 minutes. Palladium-tetrakis(triphenylphosphine) (5 mol%) was added and the reaction mixture was heated at 80 °C for 6 hours. After cooling to room temperature, the mixture was diluted with water and extracted with dichloromethane. After removing the solvent under reduced pressure the crude product was purified by column chromatography (3:1 heptane:ethyl acetate) to give 330 mg (80%) of off-white product.

^{1}H NMR (400 MHz, CHLOROFORM-D) δ 8.35 (d, *J* = 1.6 Hz, 1H), 8.15 – 8.10 (m, 2H), 8.03 (dd, *J* = 8.0, 1.7 Hz, 1H), 7.53 – 7.47 (m, 2H), 7.39 (d, *J* = 8.0 Hz, 1H), 3.95 (d, *J* = 1.5 Hz, 6H).

^{13}C NMR (101 MHz, CHLOROFORM-D) δ 166.85, 165.65, 146.04, 144.73, 134.54, 133.96, 133.77, 131.12, 129.57, 129.41, 128.65, 128.58, 122.41, 52.64, 52.39.

HRMS (ESI-TOF) Calculated for $C_{16}H_{13}BrNaO_4$ $(M+Na)^+$ 370.9895, found 370.9889

Synthesis of dimethyl 2-((trimethylsilyl)ethynyl)-[1,1'-biphenyl]-4,4'-dicarboxylate (4):

500 mg (1 eq) of **3** was dissolved in 20 mL of toluene and 10 mL of trimethylamine and degassed by bubbling nitrogen through for 10 minutes. Palladium-tetrakis(triphenylphosphine) (10 mol%) and ethynyltrimethylsilane (265 µl, 5 eq) were added and the reaction flask was sealed and heated to 75 °C for 6 hours.

After cooling to room temperature the reaction was quenched with NH_4Cl solution and extracted 3 times with dichloromethane. The organic fractions were combined and the solvent removed under reduced pressure. The crude product was purified by Kugelrohr distillation. At 80 °C triphenylphosphine oxide was removed before the temperature was increased to 150 °C to give the pure product as a waxy solid. 432 mg; 82% yield.

^{1}H NMR (400 MHz, CHLOROFORM-D) δ 8.26 (d, *J* = 1.8 Hz, 1H), 8.09 (d, *J* = 8.3 Hz, 2H), 8.03 (dd, *J* = 8.1, 1.7 Hz, 1H), 7.71 (d, *J* = 8.3 Hz, 2H), 7.46 (d, *J* = 8.1 Hz, 1H), 3.95 (d, *J* = 3.3 Hz, 6H), 0.14 (s, 9H).

^{13}C NMR (101 MHz, CHLOROFORM-D) δ 167.03, 166.25, 147.07, 143.97, 134.87, 129.79, 129.74, 129.63, 129.59, 129.46, 129.31, 122.00, 103.20, 99.49, 52.49, 52.35, -0.28.

HRMS (ESI-TOF) Calculated for $C_{21}H_{22}NaO_4Si$ $(M+Na)^+$ 389.1185 , found 389.1180

Synthesis of dimethyl 2-ethynyl-[1,1'-biphenyl]-4,4'-dicarboxylate ester (5):

4 (200 mg, 1 eq) was dissolved in dichloromethane and 5 eq of TBAF (1M, 500 µL, 5 eq) was added. The mixture was stirred for 1 hour at room temperature before the reaction was quenched with water and extracted with dichloromethane. The combined organic fractions were dried over $MgSO_4$ and the solvent was removed under reduced pressure. The crude material was purified by flash chromatography and gave the pure product as off-white solid in 103 mg (64% yield).

^{1}H NMR (400 MHz, CHLOROFORM-D) δ 8.30 (d, *J* = 1.7 Hz, 1H), 8.14 – 8.10 (m, 2H), 8.07 (dd, *J* = 8.1, 1.8 Hz, 1H), 7.67 (d, *J* = 8.6 Hz, 2H), 7.46 (s, 1H), 3.95 (d, *J* = 2.0 Hz, 6H).

HRMS (FTMS+p-NSI) Calculated for $C_{18}H_{14}NaO_4$ $(M+Na)^+$ 317.07898, found 317.07843

Synthesis of 2-ethynyl-[1,1'-biphenyl]-4,4'-dicarboxylic acid (6):

Either **4** or **5** was dissolved in a 3:2:1 mixture of THF:MeOH:H_2O and 5 eq of LiOH was added. The reaction was stirred for 2 hours before the mixture was acidified with HCl to a pH of 1. Ethyl acetate was used for extraction. The combined organic fractions were washed with brine and dried over $MgSO_4$ prior to the removal of solvent under reduced pressure. The product was obtained as light brown solid in above 90% yield.

^{1}H NMR (400 MHz, DMSO-D6) δ 8.10 (d, *J* = 1.7 Hz, 1H), 8.05 – 8.00 (m, 3H), 7.72 (d, *J* = 8.1 Hz, 2H), 7.61 (d, *J* = 8.1 Hz, 1H), 4.30 (s, 1H).

^{13}C NMR (101 MHz, DMSO-D6) δ 167.04, 166.28, 142.95, 134.25, 130.72, 130.55, 130.08, 129.91, 129.22, 129.18, 120.33, 84.74, 81.67.

HRMS (FTMS+p-NSI) Calculated for $C_{16}H_9O_4$ $(M-H)^-$ 265.05063, found 265.05032

Synthesis of dimethyl 2-nitro-[1,1'-biphenyl]-4,4'-dicarboxylate (8):

Method adapted from the literature.[3] Dimethyl-[1,1'-biphenyl]-4,4'-dicarboxylate (1.5 g, 1 eq) was dissolved in 20 mL of concentrated H_2SO_4 at room temperature before being cooled to –30 °C. 0.9 eq of 1/2 concentrated nitric acid in 2 mL H_2SO_4 was added drop wise under vigorous stirring at below –20°C. The mixture was kept at this temperature for two hours before the reaction mixture was poured onto crushed ice. Upon melting of the ice the precipitate was separated by filtration and washed thoroughly with water to remove residue acid. The crude product was purified by recrystallization from isopropanol to give **8** as white solid in 76% yield.

^{1}H NMR (400 MHz, CHLOROFORM-D) δ 8.55 (d, *J* = 1.5 Hz, 1H), 8.31 – 8.27 (m, 1H), 8.11 (dd, *J* = 8.4, 1.8 Hz, 2H), 7.54 (d, *J* = 8.0 Hz, 1H), 7.40 (dd, *J* = 8.4, 1.8 Hz, 2H), 4.00 (s, *J* =

1.0 Hz, 3H), 3.95 (s, *J* = 0.9 Hz, 3H).^{13}C NMR (101 MHz, CHLOROFORM-D) δ 166.58, 164.84, 149.09, 141.19, 139.62, 133.24, 132.23, 131.17, 130.64, 130.18, 128.01, 125.63, 52.99, 52.45. HRMS (ESI-TOF) Calculated for $C_{16}H_{13}NNaO_6$ $(M+Na)^+$ 338.0641, found 338.0635

Synthesis of dimethyl 2-amino-[1,1'-biphenyl]-4,4'-dicarboxylate (9):

Method adapted from the literature.[4] 2.15 g of **8** (1 eq) was dissolved in 10 mL of freshly distilled THF in a round bottom flask. After adding 20 mol% of palladium on carbon the flask was sealed and put under a hydrogen atmosphere using a balloon. The reaction mixture was stirred at room temperature until the TLC showed full conversion of the starting material (2:1 pentane:EtOAc). After the reaction was complete, the catalyst was filtered off *via* a celite plug and washed with fresh THF. The solvent was removed under reduced pressure to give 1.65 g of yellow solid (85% yield).

^{1}H NMR (400 MHz, $CDCl_3$) δ 8.13 (d, *J* = 8.3 Hz, 2H), 7.55 (d, *J* = 8.3 Hz, 2H), 7.51 – 7.42 (m, 2H), 7.18 (d, *J* = 7.9 Hz, 1H), 3.95 (s, 3H), 3.92 (s, 3H).

^{13}C NMR (101 MHz, $CDCl_3$) δ 167.14, 166.87, 143.64, 143.50, 130.87, 130.63, 130.48, 130.36, 129.59, 129.01, 119.86, 116.81, 52.38, 52.28, 30.46, 29.84.

HRMS (ESI-TOF) Calculated for $C_{16}H_{15}NO_4Na$ $(M+Na)^+$ 308.0899, found 308.0893

Synthesis of dimethyl 2-azido-[1,1'-biphenyl]-4,4'-dicarboxylate (10):

300 mg (1 eq) of **9** was dissolved in 3 mL of THF followed by the addition of 188 µL *t*-butyl nitrite (1.5 eq) and TMS-azide (209 µl, 1.5 eq). The reaction mixture was stirred at room temperature over night before it was quenched with water and extracted with ethyl acetate tree times. The combined organic fractions were extracted with brine and dried over $MgSO_4$ before the solvent was removed under reduced pressure to give the product in quantitative yield as a yellow solid.

^{1}H NMR (400 MHz, CHLOROFORM-D) δ 8.11 (dd, *J* = 6.8, 1.3 Hz, 2H), 7.94 (d, *J* = 1.3 Hz, 1H), 7.88 (dd, *J* = 8.0, 1.4 Hz, 1H), 7.59 – 7.52 (m, 2H), 7.42 (dd, *J* = 8.0, 0.8 Hz, 1H), 3.96 (dd, *J* = 9.9, 1.1 Hz, 6H).

^{13}C NMR (101 MHz, CHLOROFORM-D) δ 166.88, 166.07, 141.89, 137.95, 131.36, 129.62, 129.58, 126.18, 120.08, 52.66, 52.39.

HRMS (ESI-TOF) Calculated for $C_{16}H_{13}N_3NaO_4$ $(M+Na)^+$ 334.0804, found 334.0798

Synthesis of 2-azido-[1,1'-biphenyl]-4,4'-dicarboxylic acid (11):

Method adapted from the literature.[5] 1 eq (300 mg) of **10** was dissolved in a 3:2:1 mixture of THF:MeOH:H_2O at room temperature. LiOH (5 eq., 115 mg) was added and the mixture was

stirred overnight. The reaction mixture was next acidified with 1M HCl to a pH of about 1, extracted three times with EtOAc and the combined organic phases washed once with brine. The combined organic phases were dried over $MgSO_4$ before the solvent was removed under reduced pressure to give the product as yellow solid in quantitative yield.

^{1}H NMR (400 MHz, DMSO-D6) δ 8.00 (d, *J* = 8.1 Hz, 2H), 7.84 – 7.78 (m, 2H), 7.62 (d, *J* = 8.1 Hz, 2H), 7.54 (d, *J* = 7.9 Hz, 1H).

^{13}C NMR (101 MHz, DMSO-D6) δ 167.73, 166.96, 141.64, 137.62, 136.19, 132.40, 131.87, 130.60, 130.06, 129.71, 126.48, 120.23.

HRMS (FTMS+p-NSI) Calculated for $C_{14}H_8N_3O_4^-$ $(M-H)^-$ 282.05203, found 282.05184

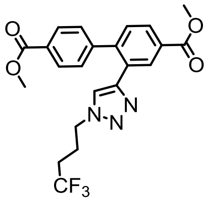

Synthesis of dimethyl 2-(1-(4,4,4-trifluorobutyl)-1H-1,2,3-triazol-4-yl)-[1,1'-biphenyl]-4,4'-dicarboxylate (12):

30 mg (1 eq) of **5** was dissolved in 3 ml of freshly distilled THF. 3.8 mg of tetrekis(acetonitrile)copper hexafluorophosphate (10 mol%) was added and the mixture was degassed for 10 minutes with nitrogen. After this 19 mg of **14** was added, the reaction vessel was sealed, and heated at 50 °C for 24 hours. After cooling to room temperature, the solvent was removed under reduced pressure. The residue was redissolved in EtOAc, quenched with NH_4Cl, and extracted three times with EtOAc. The combined organic fractions were washed with brine, dried over $MgSO_4$, and the solvent was removed under reduced pressure. The crude product was purified by column chromatography using 2:1 heptane:EtOAc as eluent. The product was obtained as brownish solid in 40% yield (18 mg).

^{1}H NMR (400 MHz, CHLOROFORM-D) δ 8.71 (d, *J* = 1.8 Hz, 1H), 8.07 (ddd, *J* = 8.2, 7.2, 1.7 Hz, 3H), 7.41 (d, *J* = 8.0 Hz, 1H), 7.35 – 7.31 (m, 2H), 4.28 (t, *J* = 6.5 Hz, 2H), 3.95 (d, *J* = 7.5 Hz, 6H), 2.09 – 1.92 (m, 4H).

^{13}C NMR (101 MHz, CHLOROFORM-D) δ 166.76, 166.64, 145.44, 143.67, 130.59, 130.47, 130.40, 129.91, 129.89, 129.46, 129.38, 129.34, 122.51, 52.44, 48.71, 30.60, 23.11.

HRMS (ESI-TOF) Calculated for $C_{22}H_{20}F_3N_3NaO_4$ $(M+Na)^+$ 470.1304, found 470.1298

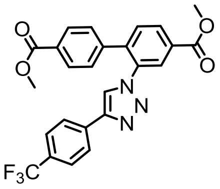

Synthesis of dimethyl 2-(4-(4-(trifluoromethyl)phenyl)-1H-1,2,3-triazol-1-yl)-[1,1'-biphenyl]-4,4'-dicarboxylate (13):

50 mg (1 eq) of **12** was dissolved in 4 mL of freshly distilled THF and tetrekis(acetonitrile)copper hexafluorophosphate (10 mol%, 6 mg) was added to the solution. The mixture was degassed for 10 minutes by bubbling nitrogen. After the addition of **15** the vessel was sealed and heated at 50 °C for 24 hours. Volatile compounds were removed under reduced pressure and the residue was redissolved in EtOAc and NH_4Cl.

Extraction with EtOAc and drying of the organic phase gave the crude product. Purification by column chromatography using 2:1 heptane:EtOAc gave 56 mg of **13** as brownish solid in 72% yield.

^{1}H NMR (400 MHz, CHLOROFORM-D) δ 8.35 (d, *J* = 1.6 Hz, 1H), 8.29 (dd, *J* = 8.1, 1.7 Hz, 1H), 7.99 (d, *J* = 8.5 Hz, 2H), 7.83 (d, *J* = 8.1 Hz, 2H), 7.66 (t, *J* = 7.6 Hz, 3H), 7.59 (s, 1H), 7.24 (s, 2H), 3.99 (s, 3H), 3.89 (s, 3H).

^{13}C NMR (101 MHz, CHLOROFORM-D) δ 166.48, 165.46, 140.93, 140.64, 131.57, 131.47, 131.31, 130.54, 130.25, 128.57, 128.14, 126.10, 122.26, 52.85, 52.46.

HRMS (ESI-TOF) Calculated for $C_{25}H_{18}F_3N_3NaO4$ $(M+Na)^+$ 504.1147, found 504.1142

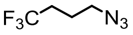

Synthesis of 4-azido-1,1,1-trifluorobutane (14)

4-bromo-1,1,1-trifluorobutane (500 mg, 1 eq) was dissolved in 7 mL of DMF and degassed for 10 minutes with nitrogen. 4 eq NaN_3 were added and the reaction mixture was stirred at room temperature overnight. The reaction was quenched with water and extracted with pentane. The combined organic fractions were washed with brine and dried over $MgSO_4$ before the solvent was carefully removed under reduced pressure without heating. The volatile product was used without further purification. Yield 295 mg; 74% as clear oil.

^{1}H NMR (400 MHz, CHLOROFORM-D) δ 3.38 (t, *J* = 6.6 Hz, 2H), 2.24 – 2.10 (m, 2H), 1.83 (ddd, *J* = 15.6, 11.0, 6.5 Hz, 2H).

^{19}F NMR (376 MHz, CHLOROFORM-D) δ -66.17, -66.20, -66.22.

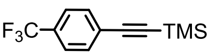

Synthesis of 1-ethynyl-4-(trifluoromethyl)benzene (15)

2 g Iodo-4-(trifluoromethyl)benzene (1 eq), bis(triphenylphosphine)palladium dichloride (340 mg, 4 mol%) and CuI (56 mg, 4 mol%) were suspended in 10 mL of distilled THF and 10 mL of Et_3N. The mixture was degassed for 10 minutes by bubbling nitrogen through the solution. After this 1.25 mL TMS-acetylene was added, the reaction vessel was sealed and heated to 40 °C for 4 hours. After cooling to room temperature the reaction was quenched with NH_4Cl and extracted with pentane. After removing the solvent under reduced pressure the product was purified by column chromatography using pentane as solvent to yield the product as off-white liquid in 69% yield (874 mg).

^{1}H NMR (400 MHz, CHLOROFORM-D) δ 7.56 (d, *J* = 0.4 Hz, 4H), 0.28 – 0.24 (m, 9H).

^{13}C NMR (101 MHz, CHLOROFORM-D) δ 132.32, 125.30, 125.26, 100.06, 97.33, -0.05.

^{19}F NMR (376 MHz, CHLOROFORM-D) δ -62.73.

Due to volatility the product was stored as TMS protected version and deprotected prior to use:

1 eq of trimethyl((4-(trifluoromethyl)phenyl)ethynyl)silane was dissolved in dichloromethane and 2 eq of TBAF (1M in DCM) was added. The mixture was stirred for 1 hour, quenched with water, dried over $MgSO_4$ and the solvent was carefully removed with an air flow.

^{1}H NMR (400 MHz, Chloroform-d) δ 7.59 (s, 4H), 3.19 (s, 1H).

^{19}F NMR (376 MHz, CHLOROFORM-D) δ -62.85.

Post synthetic linker exchange

Post synthetic linker exchange was performed on UiO–67 that was first dried under vacuum at room temperature and weighed in order to calculate the number of equivalents exchange linker needed. The resulting solid UiO-67 was suspended in the solvent mixture of choice along with the exchange linkers and gently stirred or shaken for 24 hours. After incubation, the suspension was centrifuged and the supernatant decanted. The resulting pellet was washed at least 10 times by resuspending in fresh solvent and then centrifuging down. Prior to NMR analysis the resulting MOFs were dried at room temperature under vacuum; otherwise the MOFs were stored in MeOH.

For the purposes of the click chemistry results that follow, the following abbreviations will be used:

MOF-N_3: UiO-67 containing compound **11** (L-N_3 in main text)

MOF-C≡C: UiO-67 containing compound **6** (L-C≡C in the main text)

MOF-N_3/C≡C: UiO-67 containing both **6** and **11**.

Click reactions and NMR spectra

Click reactions were performed in 2 mL microwave tubes at 50 °C in freshly distilled THF with slow stirring. In general 20 mg of UiO-67 was added to 0.5 eq tetrakis(acetonitrile)copper(I) hexafluorophosphate (calculated on the amount of clickable functionalities in the MOF) and suspended in 1.5 mL THF, degassed for 5 minutes, 2 eq. of the volatile fluorinated click partners added, and the tube sealed. The reaction was slowly stirred for 24 hours. After this time, all the volatile compounds were removed with a flow of inert gas and the MOF was further dried in vacuum. For NMR analysis about 5 mg of the resulting MOF was digested in an NMR tube containing d6-DMSO and 5 µL aqueous HF.

Table S1. Click control reactions of UiO-67 and UiO-67 modified by post synthetic exchange to include L-N_3 and L-C≡C appended linkers. Clicked products detected by ^{1}H NMR of post-reaction digested MOF.

double click reactions				
modified linkers?	step	cat.	reactants	observed clicked products
L-N_3 & L-C≡C	1)	-	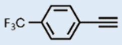	none
	2)	-	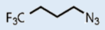	
L-N_3 & L-C≡C	1)	Cu^I	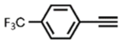	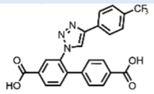
	2)	Cu^I	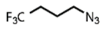	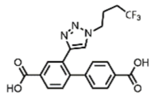
L-N_3 & L-C≡C	1)	Cu^I	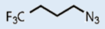	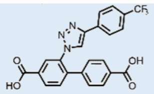
	2)	Cu^I	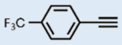	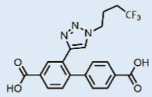

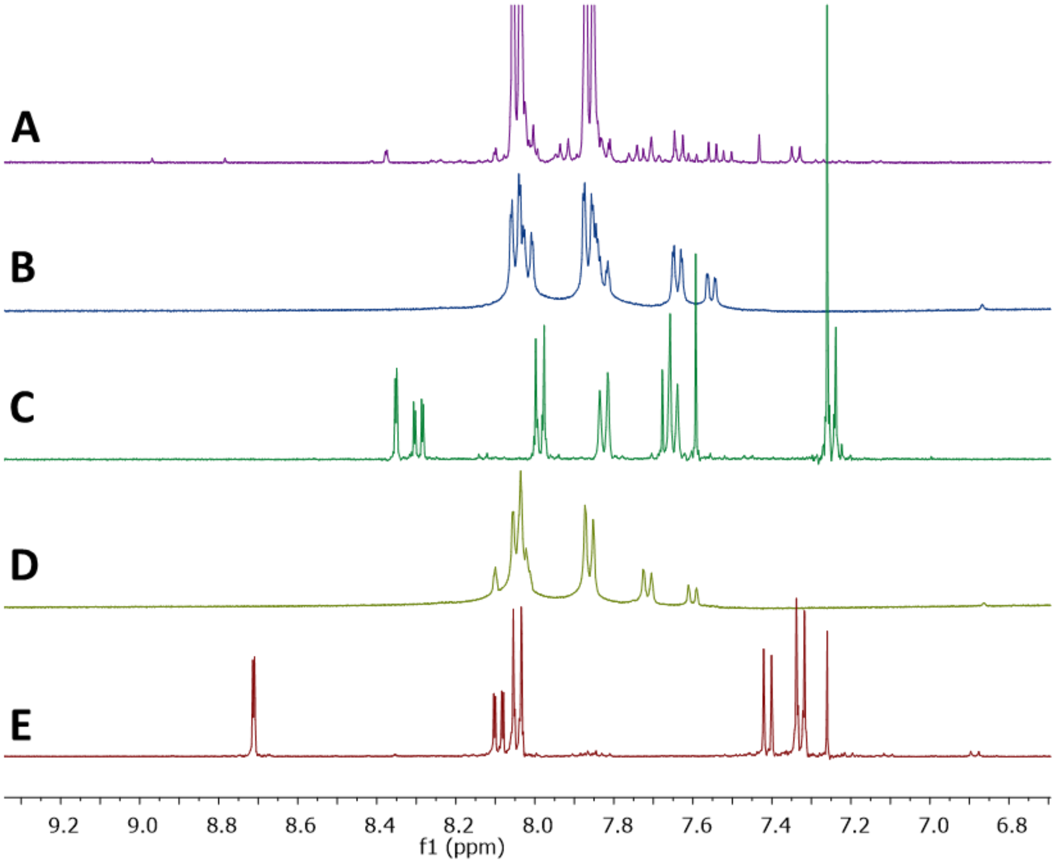

Figure S1. Typical ^{1}H NMR (d_6-DMSO) of **A)** a double clicked MOF after digestion in comparison **B) MOF-N$_3$** after digestion, **C)** reference material **13**, **D) MOF-C≡C** after digestion, and **E)** reference material **12**.

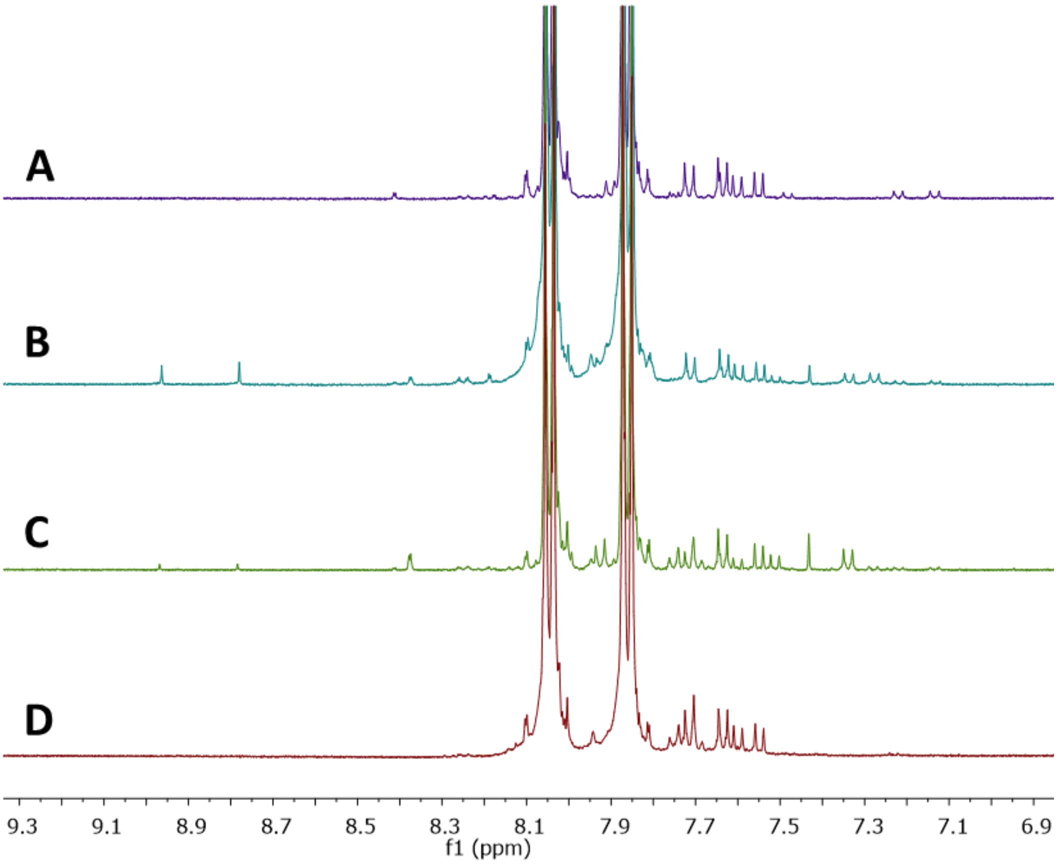

Figure S2. ^{1}H NMR (d_6-DMSO) of digested **MOF-N_3/C≡C** after click reactions. **A)** Cu catalyst, no click partners. **B)** Cu catalyst with 1-ethynyl-4-(trifluoromethyl)benzene first, then Cu catalyst with 4-azido-1,1,1-trifluorobutane**. C)** Cu catalyst with 4-azido-1,1,1-trifluorobutane first, then Cu catalyst with 1-ethynyl-4-(trifluoromethyl)benzene**. D)** only 1-ethynyl-4-(trifluoromethyl)benzene first, then only 4-azido-1,1,1-trifluorobutane.

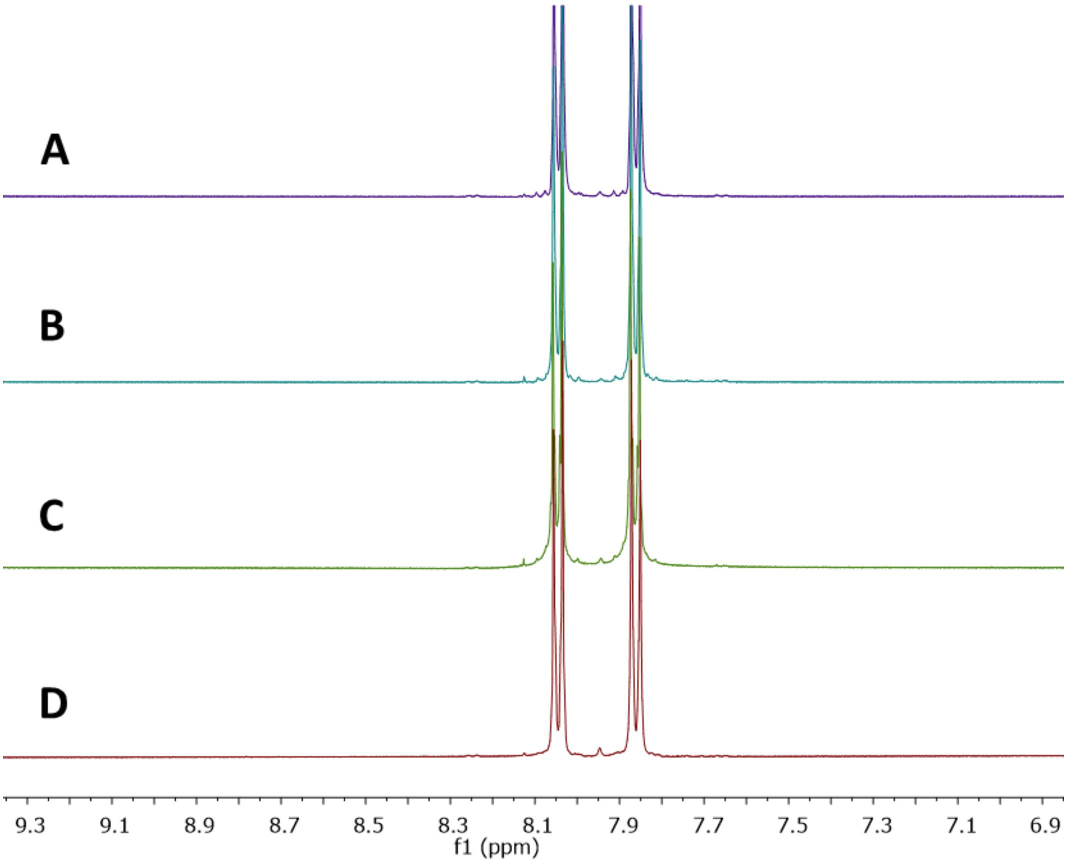

Figure S3. ^{1}H NMR (d_6-DMSO) of digested UiO-67 after click reactions. **A)** just Cu catalyst. **B)** only 1-ethynyl-4-(trifluoromethyl)benzene. **C)** only 4-azido-1,1,1-trifluorobutane. **D)** Cu catalyst with 1-ethynyl-4-(trifluoromethyl)benzene first, then Cu catalyst with 4-azido-1,1,1-trifluorobutane.

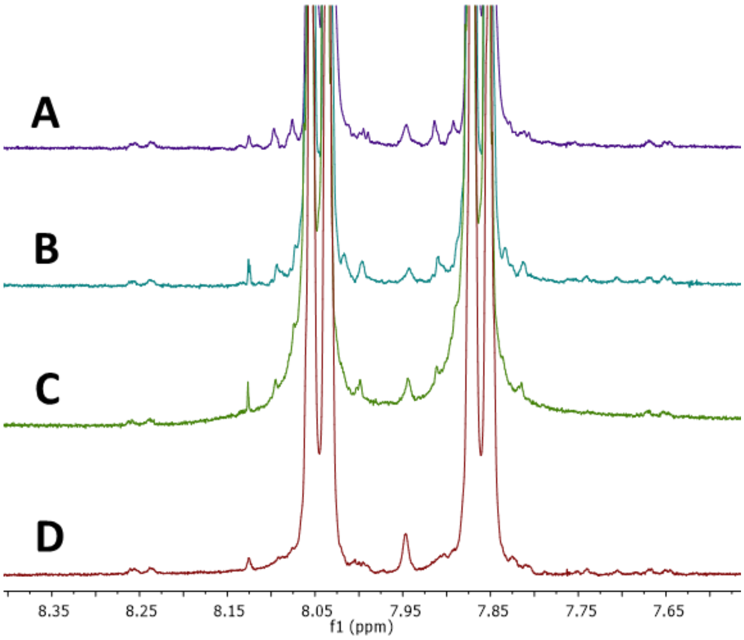

Figure S4. Enlargement of **Figure S3**.

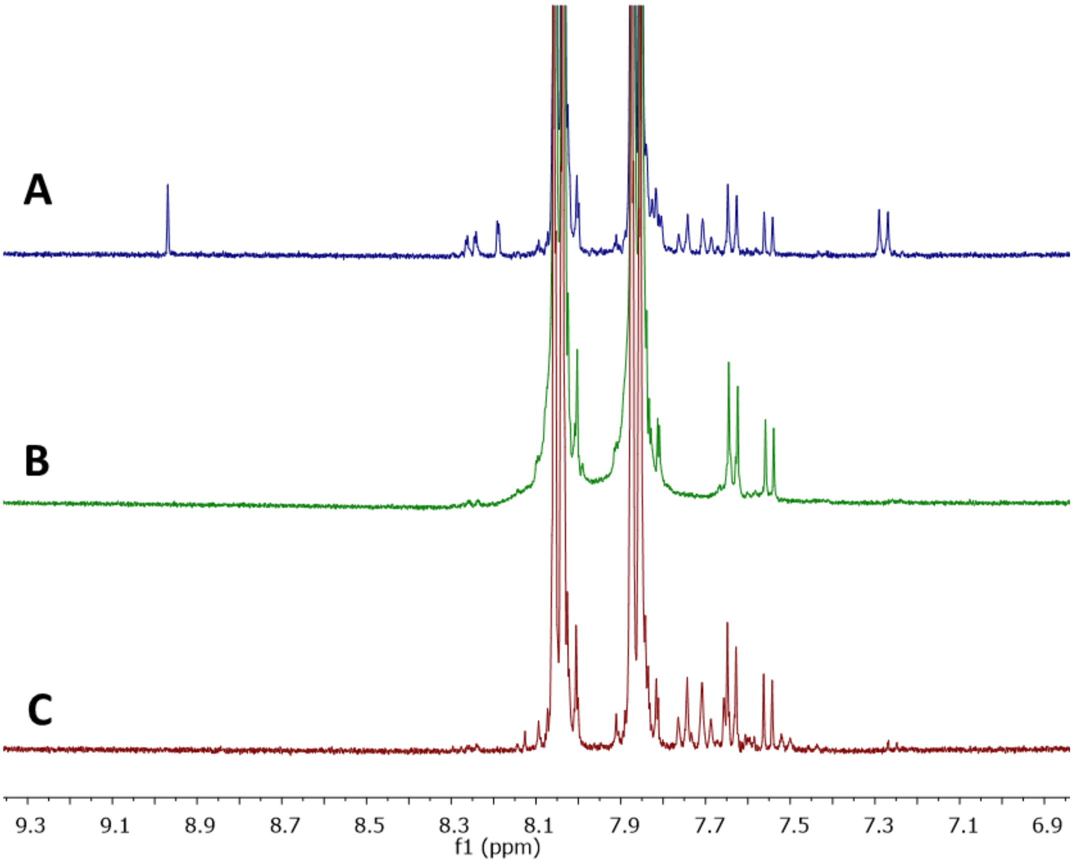

Figure S5. ^{1}H NMR (d_6-DMSO) of digested **MOF-N$_3$** after click reactions. **A)** Cu catalyst with 1-ethynyl-4-(trifluoromethyl)benzene. **B)** only Cu catalyst. **C)** only 1-ethynyl-4-(trifluoromethyl)benzene.

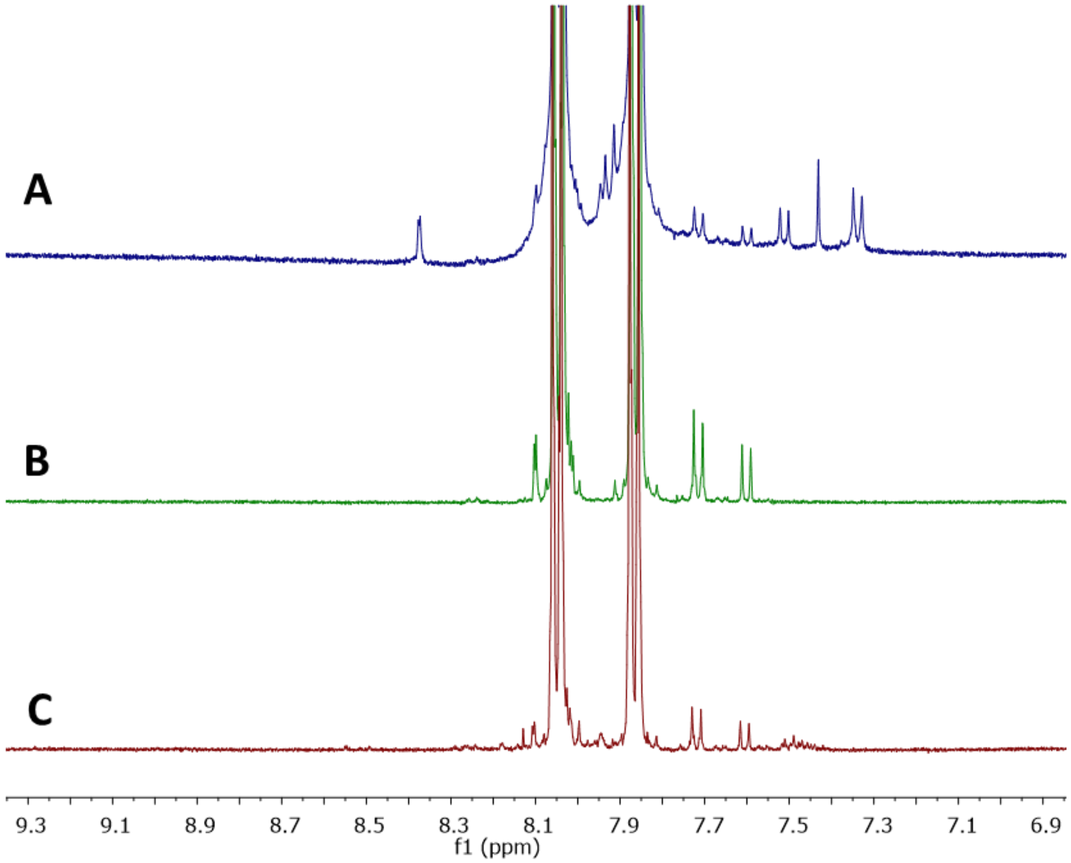

Figure S6. ^{1}H NMR (d_6-DMSO) of digested **MOF-C≡C** after click reactions. **A)** Cu catalyst with 4-azido-1,1,1-trifluorobutane. **B)** only Cu catalyst. **C)** only 4-azido-1,1,1-trifluorobutane.

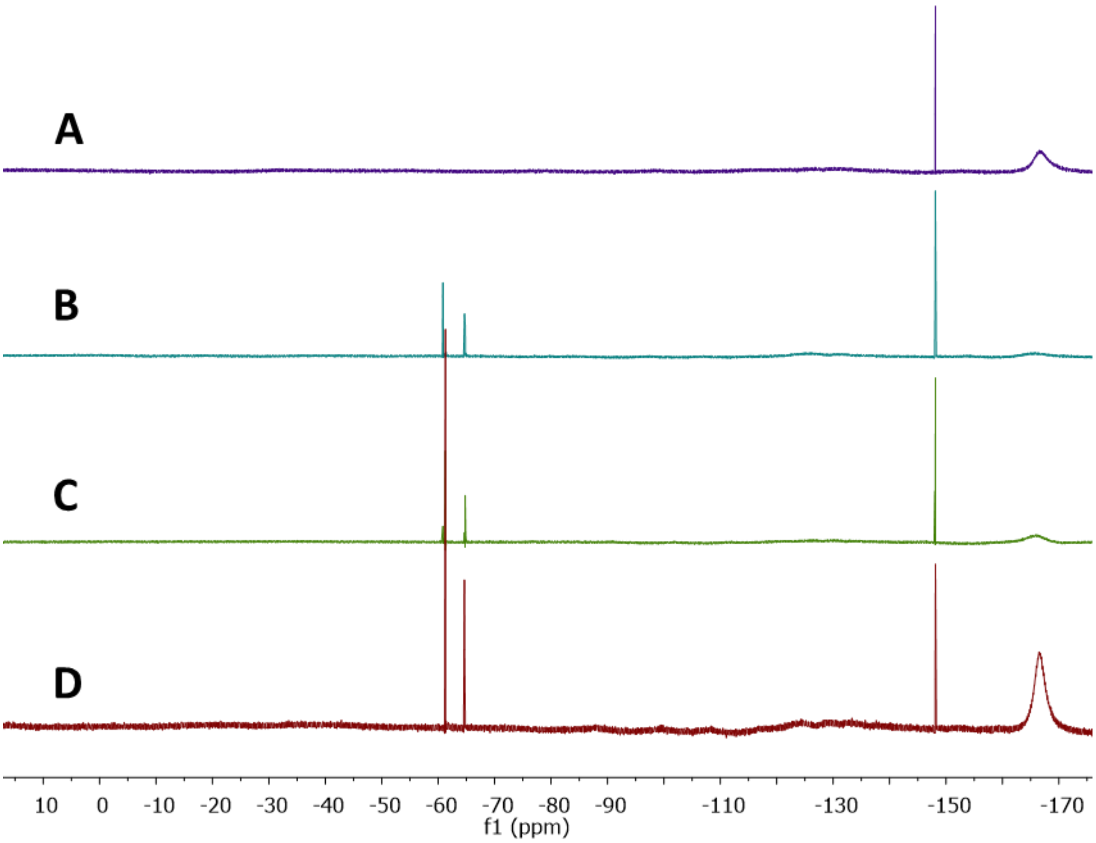

Figure S7. ^{19}F NMR (d_6-DMSO) of digested **MOF-N$_3$/C≡C** after click reactions. **A)** Cu catalyst, no click partners. **B)** Cu catalyst with 1-ethynyl-4-(trifluoromethyl)benzene first, then Cu catalyst with 4-azido-1,1,1-trifluorobutane**. C)** Cu catalyst with 4-azido-1,1,1-trifluorobutane first, then Cu catalyst with 1-ethynyl-4-(trifluoromethyl)benzene**. D)** only 1-ethynyl-4-(trifluoromethyl)benzene first, then only 4-azido-1,1,1-trifluorobutane.

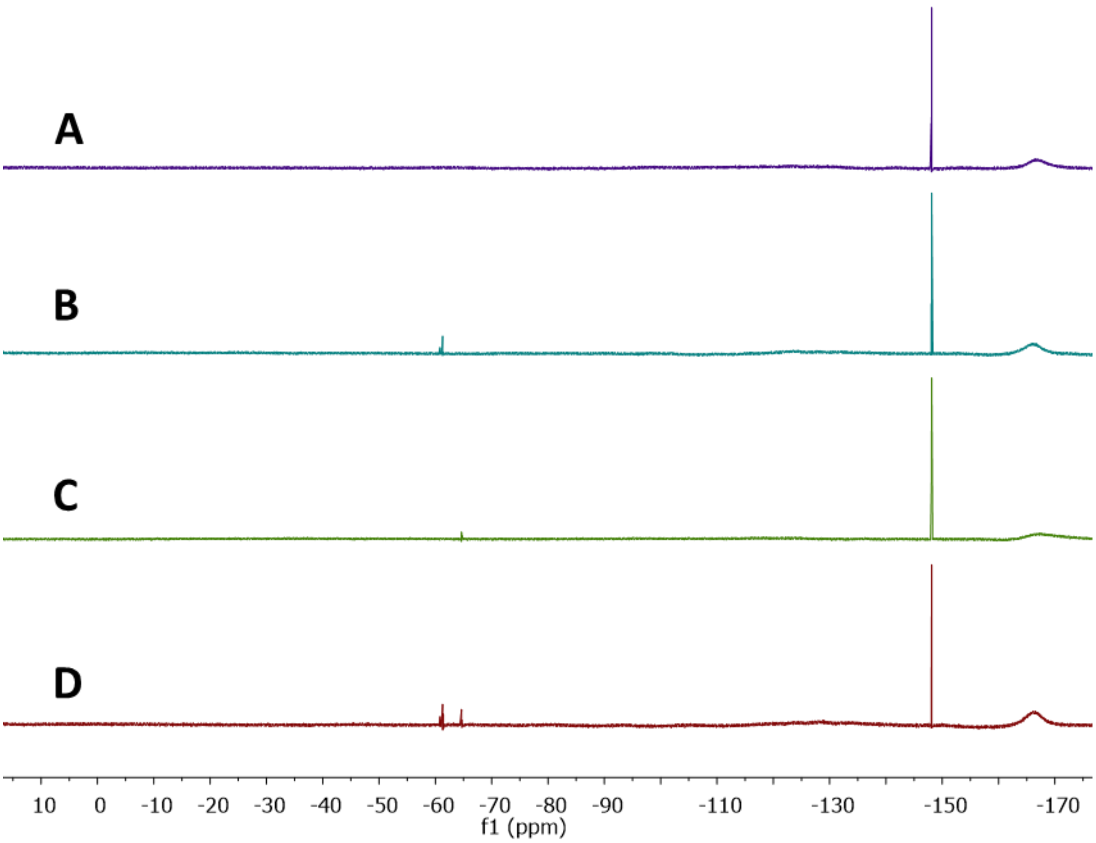

Figure S8. ^{19}F NMR (d_6-DMSO) of digested UiO-67 after click reactions. **A)** just Cu catalyst. **B)** only 1-ethynyl-4-(trifluoromethyl)benzene. **C)** only 4-azido-1,1,1-trifluorobutane. **D)** Cu catalyst with 1-ethynyl-4-(trifluoromethyl)benzene first, then Cu catalyst with 4-azido-1,1,1-trifluorobutane.

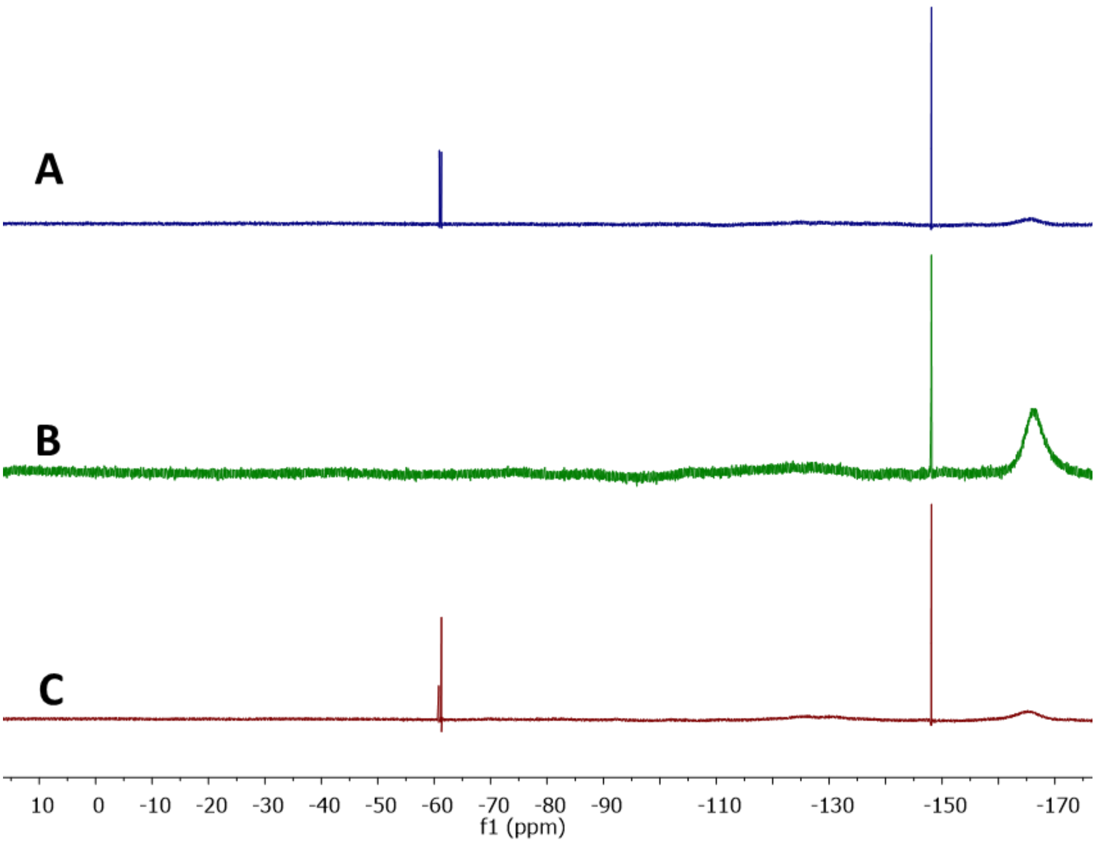

Figure S9. ^{19}F NMR (d_6-DMSO) of digested **MOF-N$_3$** after click reactions. **A)** Cu catalyst with 1-ethynyl-4-(trifluoromethyl)benzene. **B)** only Cu catalyst. **C)** only 1-ethynyl-4-(trifluoromethyl)benzene.

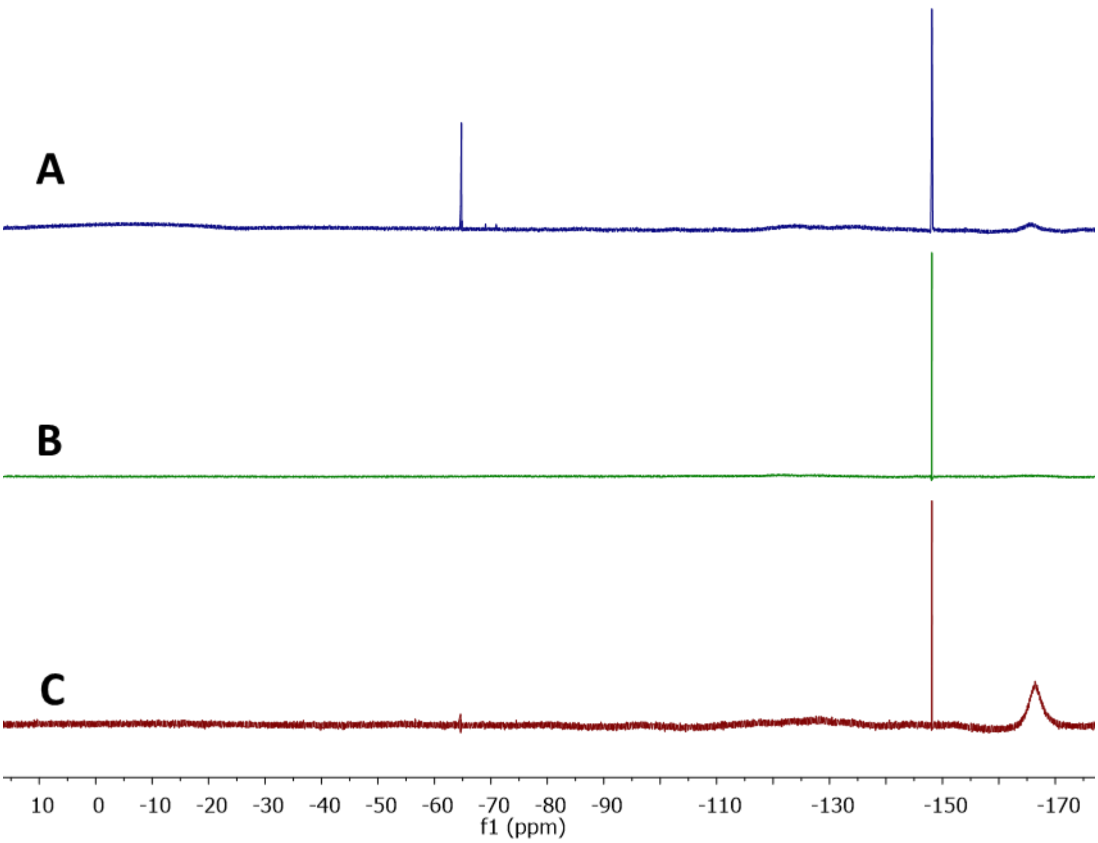

Figure S10. ^{19}F NMR (d_6-DMSO) of digested **MOF-C≡C** after click reactions. **A)** Cu catalyst with 4-azido-1,1,1-trifluorobutane. **B)** only Cu catalyst. **C)** only 4-azido-1,1,1-trifluorobutane.

Powder X-ray diffraction patterns

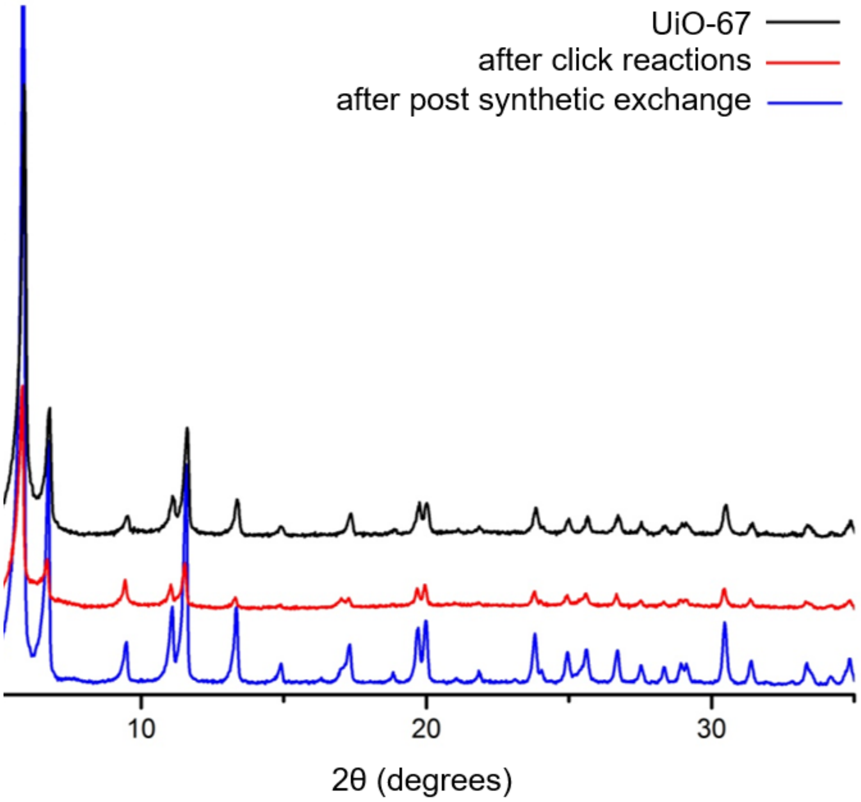

Figure S11. Powder X-ray diffraction patterns comparing native UiO-67 with **MOF-N_3/C≡C** before (blue) and after (red) the two step click reactions.

Infrared spectroscopy

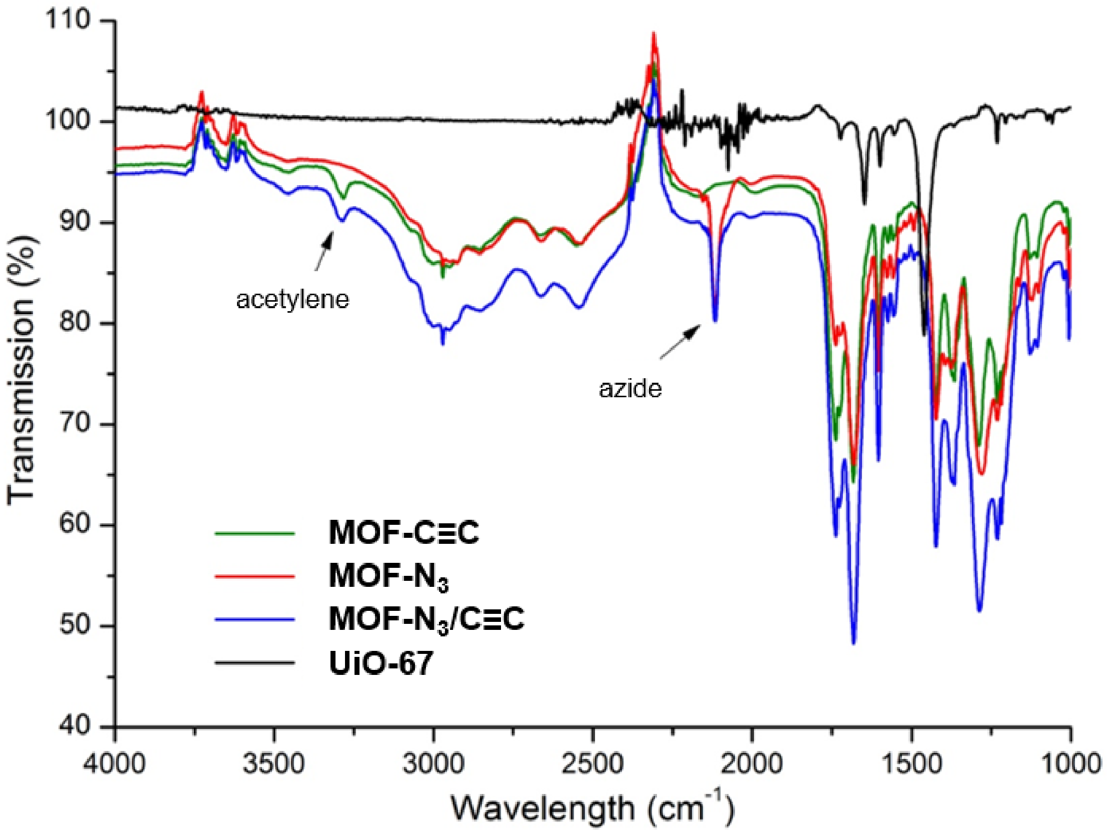

Figure S12. IR spectra of UiO-67 before and after post synthetic linker exchange using 15 mM of the exchange linkers. The most prominent peaks for acetylene (~3290) and azide (~2110) moieties are indicated.

Scanning electron microscopy

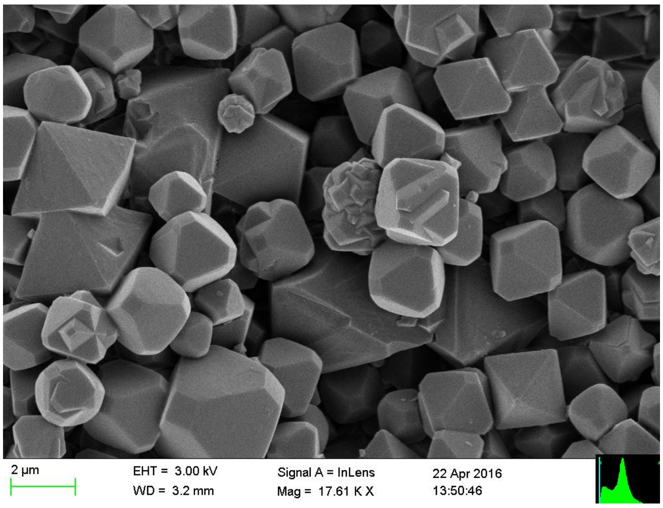

Figure S13. SEM of UiO-67 as synthesized.

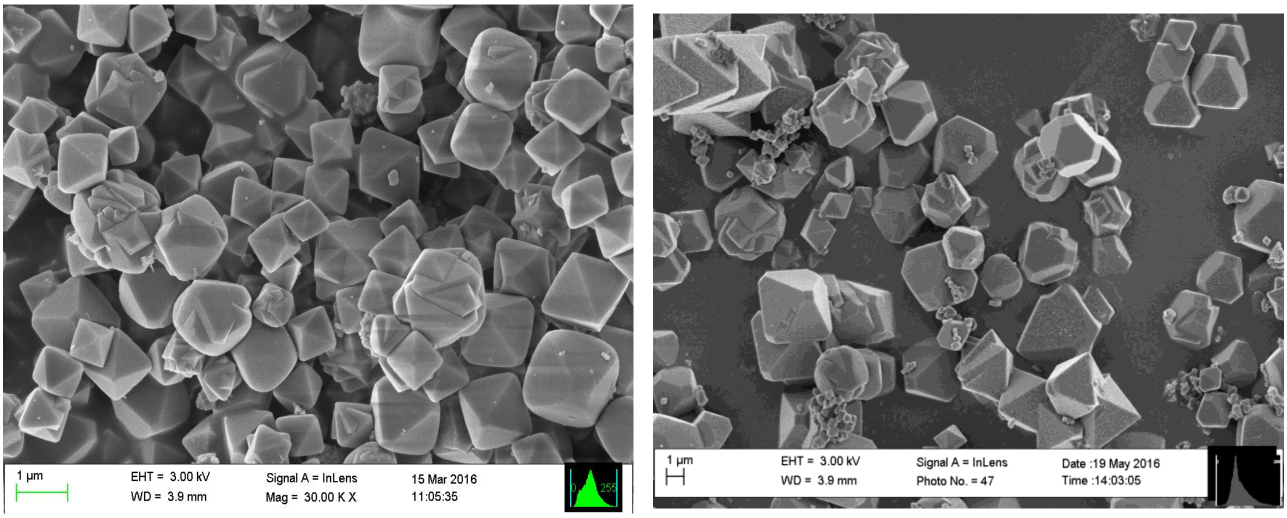

Figure S14. SEM of MOF-C≡C.

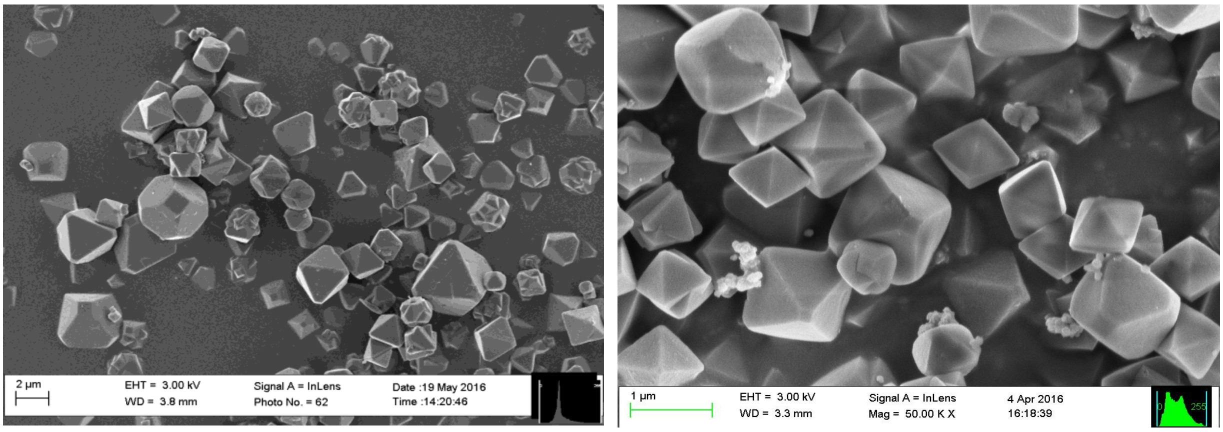

Figure S15. SEM of MOF-N_3.

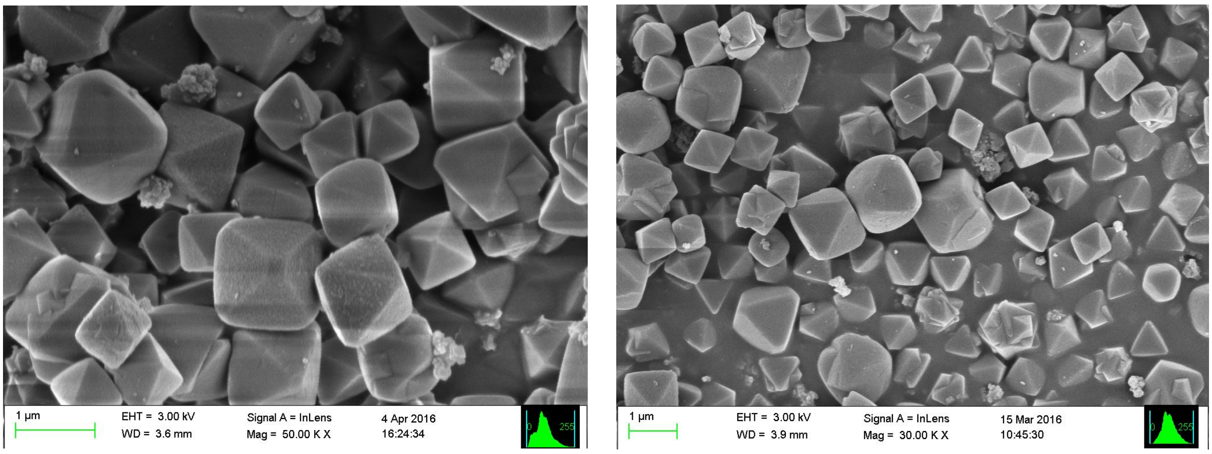

Figure S16. SEM of MOF-N_3/C≡C.

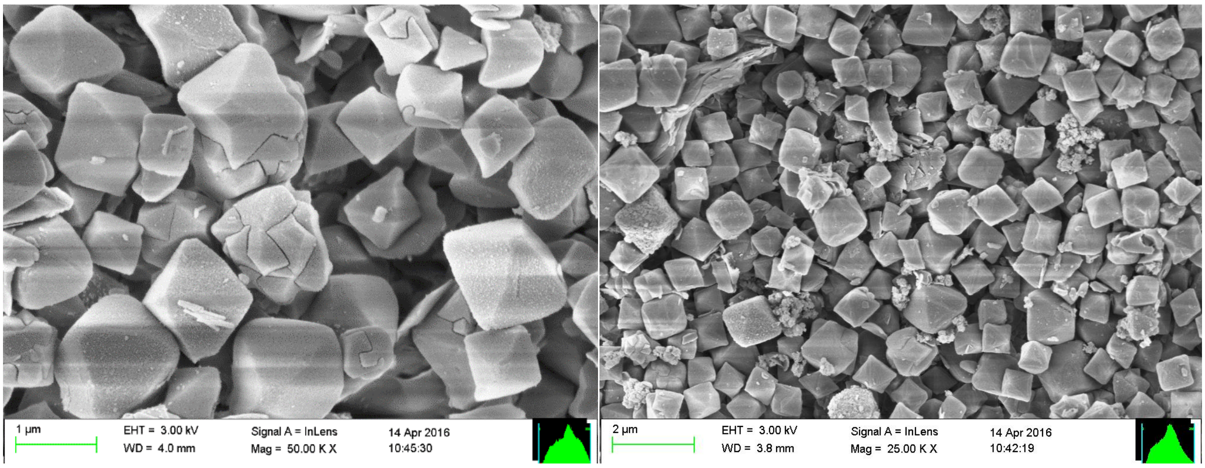

Figure S17. SEM of MOF-N_3/C≡C.

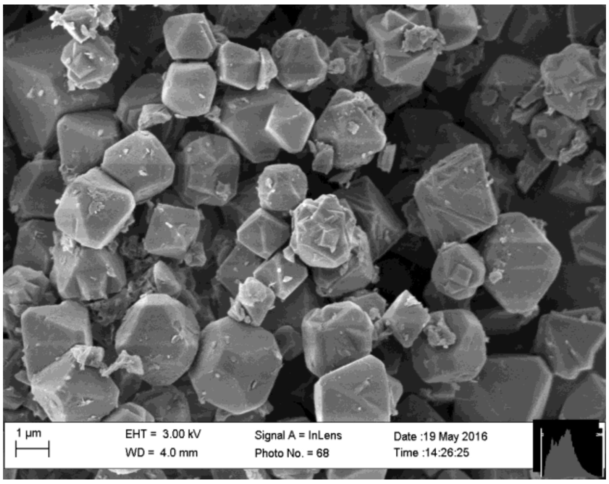

Figure S18. SEM of MOF-N_3 after the click reaction with Cu catalyst and 1-ethynyl-4-(trifluoromethyl)benzene.

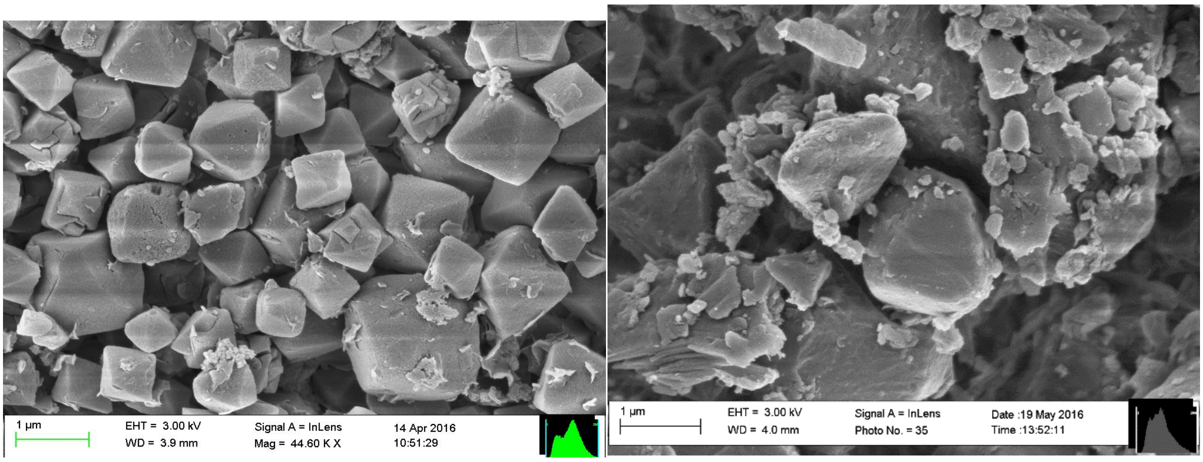

Figure S19. SEM of MOF-N_3/C≡C after the two-step click reactions using Cu catalyst and stirring. The right picture shows a magnification of the crystal damage.

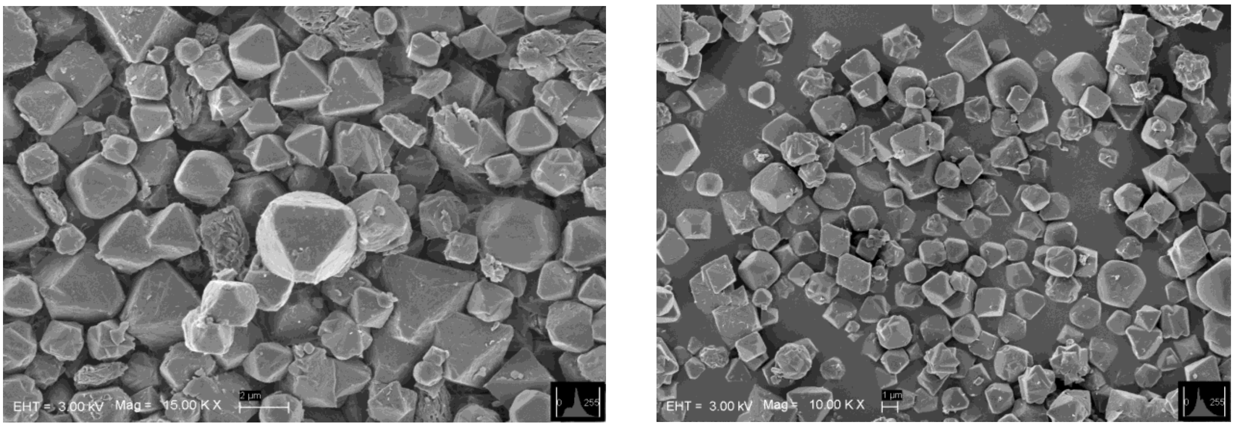

Figure S20. SEM of MOF-N_3/C≡C after the two-step click reactions using Cu catalyst and shaking instead of stirring. Less damage is visible.

References

1 G. R. Fulmer, A. J. M. Miller, N. H. Sherden, H. E. Gottlieb, A. Nudelman, B. M. Stoltz, J. E. Bercaw and K. I. Goldberg, *Organometallics*, 2010, **29**, 2176–2179.

2 A. Schaate, P. Roy, A. Godt, J. Lippke, F. Waltz, M. Wiebcke and P. Behrens, *Chem. - A Eur. J.*, 2011, **17**, 6643–6651.

3 C. Kutzscher, H. C. Hoffmann, S. Krause, U. Stoeck, I. Senkovska, E. Brunner and S. Kaskel, *Inorg. Chem.*, 2015, **54**, 1003–1009.

4 D. J. Lun, G. I. N. Waterhouse and S. G. Telfer, *J. Am. Chem. Soc.*, 2011, **133**, 5806–5809.

5 C. Liu, T. Li and N. L. Rosi, *J. Am. Chem. Soc.*, 2012, **134**, 18886–18888.
